# BLM and RMI1 Alleviate RPA Inhibition of TopoIIIα Decatenase Activity

**DOI:** 10.1371/journal.pone.0041208

**Published:** 2012-07-20

**Authors:** Jay Yang, Csanad Z. Bachrati, Ian D. Hickson, Grant W. Brown

**Affiliations:** 1 Department of Biochemistry and Donnelly Centre for Cellular and Biomolecular Research, University of Toronto, Toronto, Ontario, Canada; 2 Weatherall Institute of Molecular Medicine, University of Oxford, John Radcliffe Hospital, Oxford, United Kingdom; 3 Center for Healthy Aging, Department of Cellular and Molecular Medicine, University of Copenhagen, Copenhagen, Denmark; Saint Louis University, United States of America

## Abstract

RPA is a single-stranded DNA binding protein that physically associates with the BLM complex. RPA stimulates BLM helicase activity as well as the double Holliday junction dissolution activity of the BLM-topoisomerase IIIα complex. We investigated the effect of RPA on the ssDNA decatenase activity of topoisomerase IIIα. We found that RPA and other ssDNA binding proteins inhibit decatenation by topoisomerase IIIα. Complex formation between BLM, TopoIIIα, and RMI1 ablates inhibition of decatenation by ssDNA binding proteins. Together, these data indicate that inhibition by RPA does not involve species-specific interactions between RPA and BLM-TopoIIIα-RMI1, which contrasts with RPA modulation of double Holliday junction dissolution. We propose that topoisomerase IIIα and RPA compete to bind to single-stranded regions of catenanes. Interactions with BLM and RMI1 enhance toposiomerase IIIα activity, promoting decatenation in the presence of RPA.

## Introduction

BLM is a member of the RecQ family of helicases that safeguards genome integrity [1]. Bi-allelic mutations in the *BLM* gene give rise to Bloom syndrome, a rare cancer predisposition disorder in humans [2]. The BLM core complex, composed of BLM, DNA topoisomerase IIIα (TopoIIIα), and RMI1, is a suppressor of illegitimate recombination [3]. This complex catalyses a remarkable dissolution reaction that leads to the dissociation of DNA structures containing two Holliday junctions into genetically silent non-crossover products [4]–[8]. The double Holliday junction (DHJ) dissolution reaction consists of two enzymatic steps: 1) branch migration of two Holliday junctions towards each other by the helicase activity of BLM and the relaxation activity of TopoIIIα, resulting in two duplex DNAs interlinked via catenated single strands, and 2) unlinking of the resulting structure, termed a hemicatenane, by the decatenase activity of TopoIIIα [4], [9]. Because DHJs resemble intermediates that arise from the process of homologous recombination, the dissolution activity of BLM-TopoIIIα-RMI1 provides a clear explanation of why cells from BS patients exhibit genetic instability associated with elevated levels of sister-chromatid exchanges [4], [10], [11].

Replication Protein A (RPA) is a single-stranded DNA (ssDNA) binding protein that is indispensable in all eukaryotes [12]. RPA plays essential roles in many aspects of DNA metabolism processes including DNA replication, DNA repair, recombination, and DNA damage checkpoint signaling [13]. RPA homologs, which are highly conserved among eukaryotic organisms [14], are heterotrimeric complexes composed of subunits of 70-, 32-, and 14-kDa in size [15], [16]. Members of this family bind non-specifically to single-stranded DNA with high affinity via four conserved oligonucleotide-binding folds (OB-folds) [17]. The binding of ssDNA by RPA follows a hierarchical assembly pathway in which OB-folds bind sequentially from the 5′ to 3′ direction on ssDNA [17]. Naked ssDNA is a source of genome instability because of its tendency to form secondary structures and its susceptibility to nucleolytic cleavage [18], [19]. Therefore, RPA maintains genome integrity by binding to and protecting ssDNA until DNA metabolism processes are complete.

RPA associates with the BLM complex. RPA co-immunoprecipitates with BLM and RMI1 [20], [21] and specifically stimulates the DHJ dissolution activity of BLM-TopoIIIα [5]. RPA directly interacts with BLM helicase via its 70 kDa subunit in a manner that is independent of DNA [22]. RPA inhibits BLM strand-annealing activity while specifically stimulating BLM helicase activity to unwind long stretches of duplex DNA [22], [23]. The stimulation requires the physical interaction between BLM and RPA [24], and is diminished when RPA is replaced with *E. coli* SSB (Single-stranded Binding Protein) (EcSSB) [25]. Therefore, RPA enhances BLM activity to unwind double-stranded DNA by two distinct mechanisms; RPA not only passively prevents the re-annealing of unwound ssDNA, but also actively promotes duplex DNA unwinding via a direct protein-protein interaction. Together, these data argue that the stimulation of DHJ dissolution by RPA is in part due to the specific stimulation of BLM helicase activity.

In this study, we investigated whether RPA modulates the second step of the dissolution reaction, the decatenation by TopoIIIα. Using a previously established *in vitro* system that mimics the latest stage in DHJ dissolution [26] we found that RPA inhibits TopoIIIα decatenase activity. RPA inhibition occurs non-specifically since EcSSB also inhibits TopoIIIα decatenase activity. Interestingly, BLM alleviates the inhibition of TopoIIIα decatenase activity by either RPA or EcSSB. However, BLM does not alleviate the inhibition of EcTop1 decatenase activity by EcSSB or RPA, suggesting that the specific interaction between BLM and TopoIIIα, but not between TopoIIIα and RPA, is crucial for TopoIIIα action on RPA- (or EcSSB-) coated single-stranded DNA substrates. Together, these data indicate the complex nature of the interplay among BLM core complex members during the steps of DHJ dissolution.

**Figure 1:**
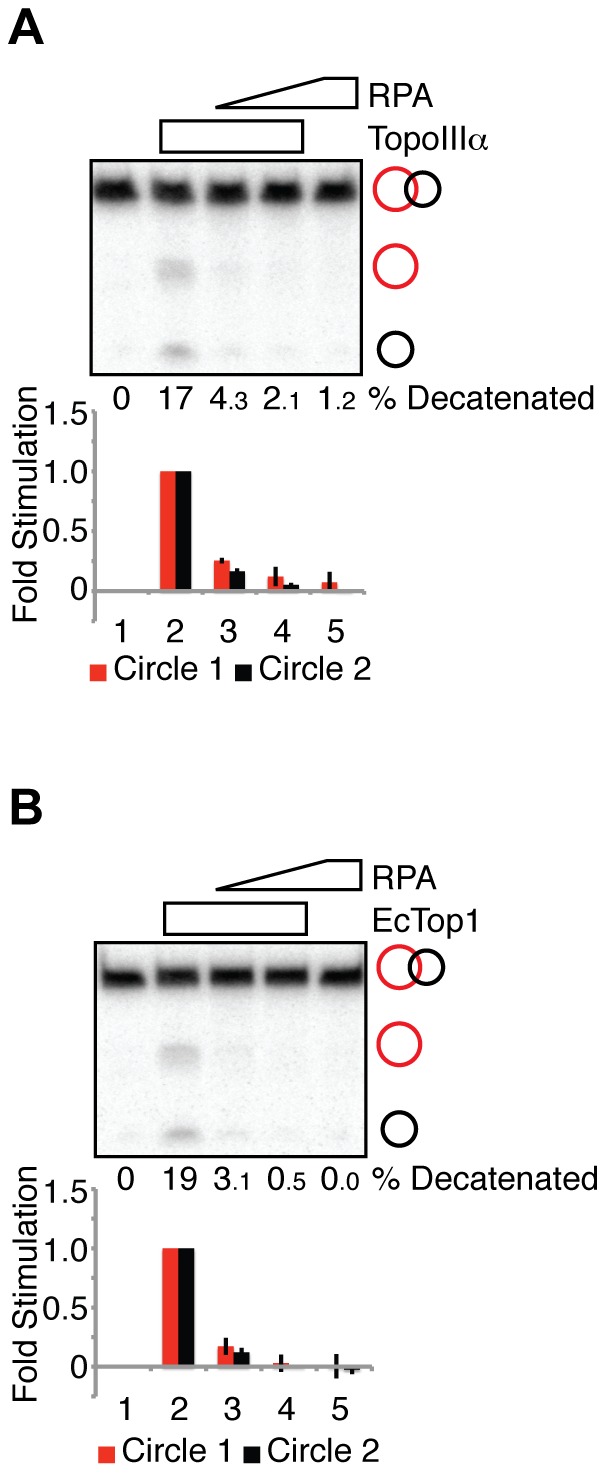
RPA inhibits the decatenase activity of both TopoIIIα and EcTop1. (**A**) Decatenation reactions containing TopoIIIα (30 nM, lanes 2–4) and RPA (100 nM, lane 3; 200 nM, lanes 4 and 5) as indicated were fractionated on a denaturing polyacrylamide gel and autoradiographed. Quantification of the decatenation products is presented in the histogram, normalized to the reactions in lane 2 (TopoIIIα alone). The percent of catenated substrate converted to circular products is indicated. (**B**) Decatenation reactions containing EcTop1 (6 nM, lanes 2–4) and RPA (100 nM, lane 3; 200 nM, lanes 4 and 5) as indicated were fractionated on a denaturing polyacrylamide gel and autoradiographed. Quantification of the decatenation products is presented in the histogram, normalized to the reactions in lane 2 (EcTop1 alone). The percent of catenated substrate converted to circular products is indicated.

**Figure 2:**
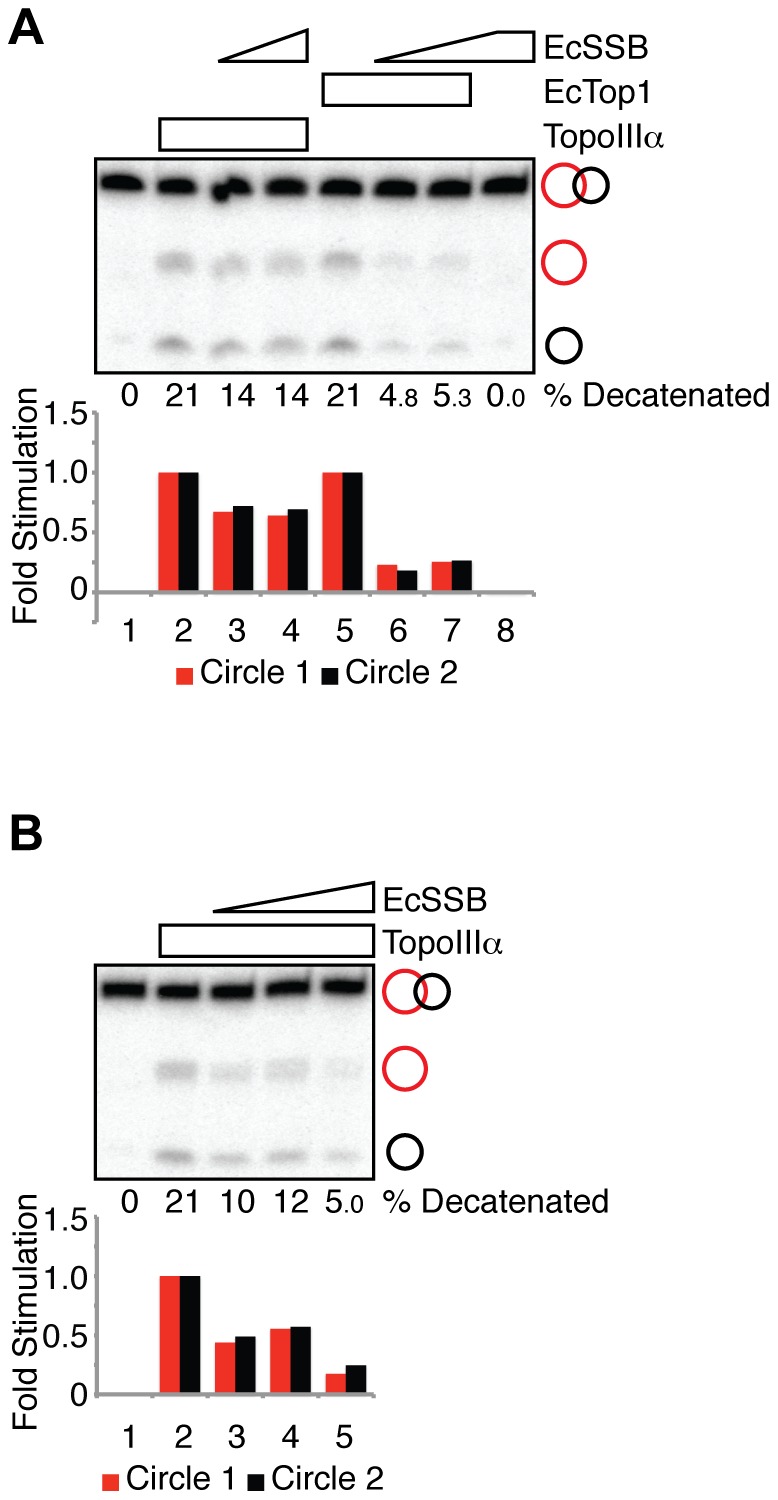
EcSSB inhibits the decatenase activity of both TopoIIIα and EcTop1. (**A**) Decatenation reactions containing TopoIIIα (30 nM, lanes 2–4), EcTop1 (6 nM, lanes 5–7) and EcSSB (100 nM, lanes 3 and 6; 200 nM, lanes 4, 7 and 8) as indicated were fractionated on a denaturing polyacrylamide gel and autoradiographed. Quantification of the decatenation products is presented in the histogram, normalized to the reactions in lane 2 (TopoIIIα alone) or lane 5 (EcTop1 alone). The percent of catenated substrate converted to circular products is indicated. (**B**) Decatenation reactions containing TopoIIIα (30 nM, lanes 2–5) and EcSSB (0.4 mM, lane 3; 0.8 mM, lane 4; 3.2 mM, lane 5) as indicated were fractionated on a denaturing polyacrylamide gel and autoradiographed. Quantification of the decatenation products is presented in the histogram, normalized to the reactions in lane 2 (TopoIIIα alone). The percent of catenated substrate converted to circular products is indicated.

**Figure 3:**
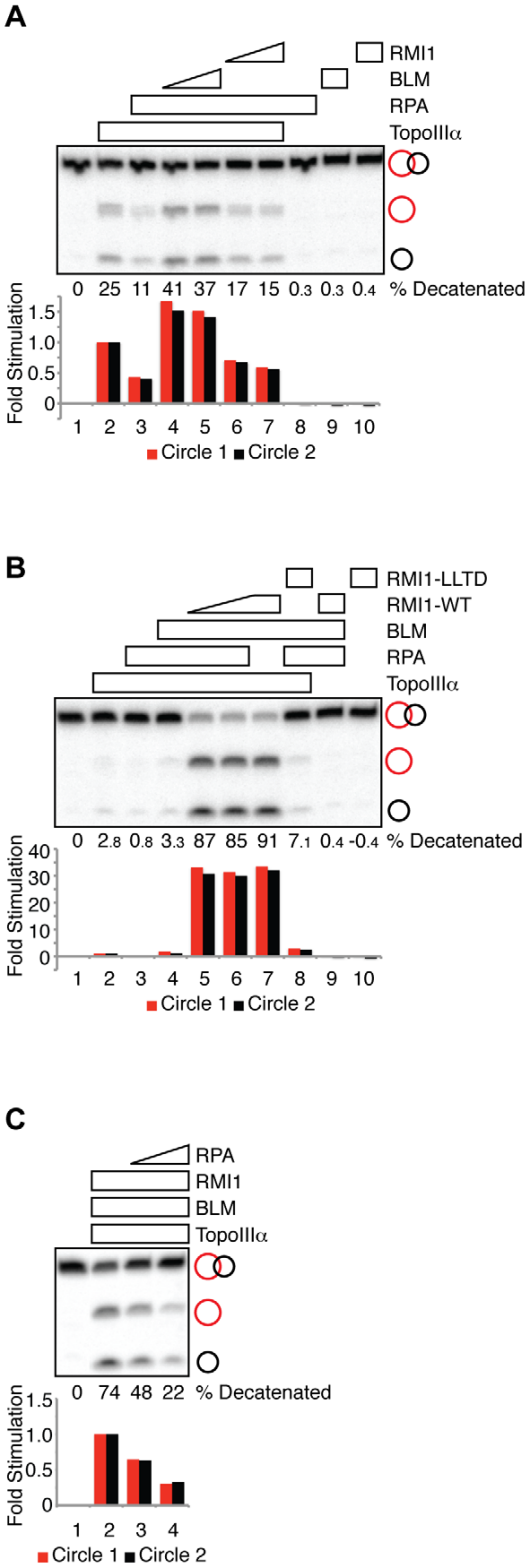
BLM-RMI1 alleviates RPA inhibition of TopoIIIα decatenase activity. (**A**) Decatenation reactions containing TopoIIIα (30 nM, lanes 2–7), RPA (100 nM, lanes 3–8), BLM (33 nM, lane 4; 66 nM, lanes 5 and 9) and RMI1 (100 nM, lane 6; 200 nM, lanes 7 and 10) as indicated were fractionated on a denaturing polyacrylamide gel and autoradiographed. Quantification of the decatenation products is presented in the histogram, normalized to the reactions in lane 2 (TopoIIIα alone). The percent of catenated substrate converted to circular products is indicated. (**B**) Decatenation reactions containing TopoIIIα (15 nM, lanes 2–8), RPA (100 nM, lanes 3–6, 8 and 9), BLM (17 nM, lanes 4–9), wild type RMI1 (75 nM, lane 5; 150 nM, lanes 6, 7 and 9) and RMI1-LLTD mutant (150 nM, lanes 8 and 10) as indicated were fractionated on a denaturing polyacrylamide gel and autoradiographed. Quantification of the decatenation products is presented in the histogram, normalized to the reactions in lane 2 (TopoIIIα alone). The percent of catenated substrate converted to circular products is indicated. (**C**) Decatenation reactions containing TopoIIIα (7.5 nM, lanes 2–4), BLM (8 nM, lanes 2–4), RMI1 (38 nM, lanes 2–4) and RPA (140 nM, lane 3; 280 nM, lane 4) as indicated were fractionated on a denaturing polyacrylamide gel and autoradiographed. Quantification of the decatenation products is presented in the histogram, normalized to the reactions in lane 2 (TopoIIIα-BLM-RMI1). The percent of catenated substrate converted to circular products is indicated.

**Figure 4:**
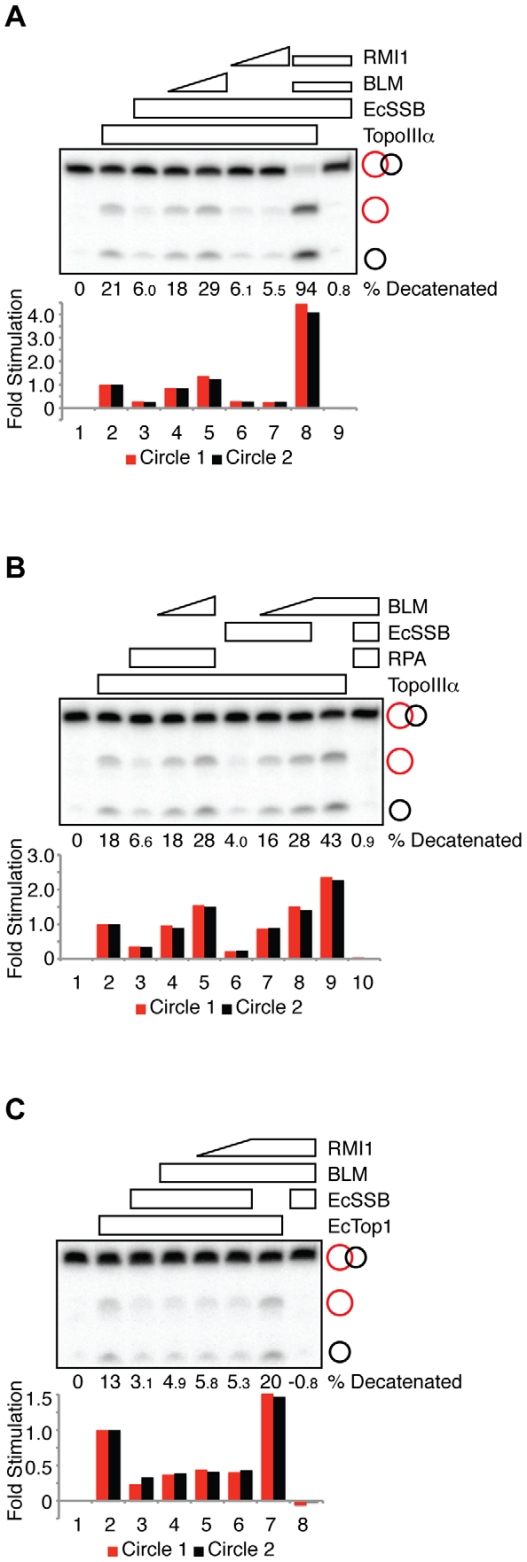
BLM alleviates EcSSB inhibition of TopoIIIα, but not EcTop1, decatenase activity. (**A**) Decatenation reactions containing TopoIIIα (30 nM, lanes 2–8), EcSSB (3.2 mM, lanes 3–9), BLM (33 nM, lanes 4, 8 and 9; 66 nM, lane 5) and RMI1 (100 nM, lanes 6, 8 and 9; 200 nM, lane 7) as indicated were fractionated on a denaturing polyacrylamide gel and autoradiographed. Quantification of the decatenation products is presented in the histogram, normalized to the reactions in lane 2 (TopoIIIα alone). The percent of catenated substrate converted to circular products is indicated. (**B**) Decatenation reactions containing TopoIIIα (20 nM, lanes 2–9), RPA (200 nM, lanes 3–5 and 10), EcSSB (3.2 mM, lanes 6–8 and 10) and BLM (33 nM, lanes 4 and 7; 66 nM, lanes 5 and 8–10) as indicated were fractionated on a denaturing polyacrylamide gel and autoradiographed. Quantification of the decatenation products is presented in the histogram, normalized to the reactions in lane 2 (TopoIIIα alone). The percent of catenated substrate converted to circular products is indicated. (**C**) Decatenation reactions containing EcTop1 (6 nM, lanes 2–7), EcSSB (100 nM, lanes 3–6 and 8), BLM (17 nM, lanes 4–8) and RMI1 (75 nM, lane 5; 150 nM, lanes 6–8) as indicated were fractionated on a denaturing polyacrylamide gel and autoradiographed. Quantification of the decatenation products is presented in the histogram, normalized to the reactions in lane 2 (EcTop1 alone). The percent of catenated substrate converted to circular products is indicated.

**Figure 5:**
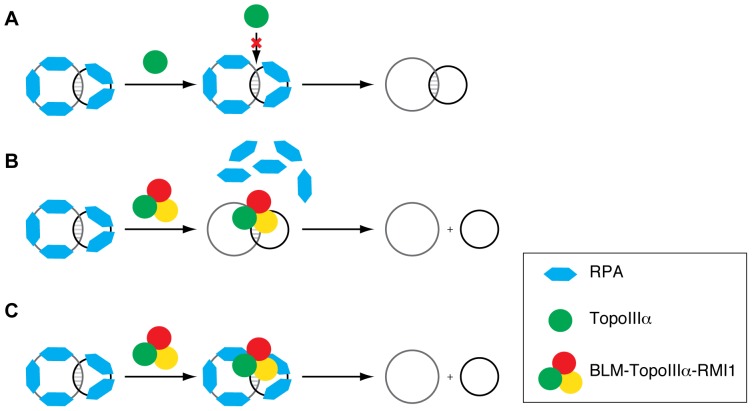
TopoIIIα, BLM and RMI1 cooperate to catalyze decatenation on RPA-coated single-stranded catenane. (**A**) In a competitive binding model, TopoIIIα alone is unable to access RPA-coated substrate. (**B**) Complex formation by BLM, TopoIIIα, and RMI1 enables displacement of RPA from the substrate. (**C**) Alternatively, the DNA binding activity of BLM drives the complex to the substrate even when the substrate is coated with RPA, promoting decatenation.

## Results

### RPA inhibits TopoIIIα decatenase activity

Because RPA positively regulates BLM-TopoIIIα-mediated DHJ dissolution [5], we asked whether RPA stimulates TopoIIIα decatenase activity. We found that TopoIIIα decatenase activity was inhibited by RPA, in a concentration dependent manner, by almost 10-fold (Figure 1A). Both TopoIIIα and *E. coli* topoisomerase I (EcTop1) belong to type IA family of topoisomerases [27]. To test the specificity of RPA inhibition, we tested the ability of RPA to inhibit EcTop1-mediated decatenation. We found that RPA reduced EcTop1-mediated decatenation at a level similar to that seen for TopoIIIα-mediated decatenation (Figure 1B). Together, these data indicate that RPA inhibits decatenation by type IA topoisomerases, and that the inhibition is likely independent of RPA-topoisomerase interactions.

### EcSSB also inhibits TopoIIIα decatenase activity

Since RPA inhibits the decatenase activities from both TopoIIIα and EcTop1, we next asked whether EcSSB, the RPA functional homolog in *E. coli*, inhibits decatenation. We incubated single-stranded catenanes with TopoIIIα or EcTop1 at concentrations at which both enzymes exhibited similar decatenase activities (Figure 2A, lane 2 versus lane 5). Upon the addition of 100 to 200 nM EcSSB to the reactions, we found that EcSSB readily inhibited EcTop1 decatenase activity by more than 4-fold (Figure 2A, lanes 6 and 7 versus lane 5). On the other hand, EcSSB at the same concentrations reduced the TopoIIIα-mediated decatenation by only 33% (Figure 2A, lanes 3 and 4 versus lane 2). Strong inhibition of TopoIIIα decatenase activity was finally achieved at 3.2 mM of EcSSB (versus 100 nM to inhibit EcTop1 activity) (Figure 2B). Thus, unlike RPA, EcSSB only efficiently inhibits the decatenase activity of EcTop1, its cognate topoisomerase. The discrepancy between the efficiency of EcSSB inhibition in each case is more likely to arise from the difference in substrate binding affinities between TopoIIIα and EcTop1, rather than as a consequence of species specific interactions, because EcTop1 and EcSSB do not physically interact with each other [28].

### BLM-RMI1 alleviates RPA inhibition of TopoIIIα decatenase activity

Since BLM and RMI1 stimulate TopoIIIα decatenase activity [26], we asked whether BLM or RMI1 alleviates RPA inhibition of TopoIIIα decatenase activity. When performing the alleviation experiment, we incubated RPA alone with single-stranded catenanes for five minutes before adding BLM, TopoIIIα or RMI1 to investigate the ability of the complex to interact with RPA-coated substrates. Interestingly, not only did BLM restore TopoIIIα activity in the presence of RPA, it also further stimulated the decatenase activity by 1.5-fold (Figure 3A, lanes 4 and 5 versus lane 2 and 3), indicating that BLM alleviates RPA inhibition of TopoIIIα-mediated decatenation. In contrast, RMI1 did not alleviate RPA inhibition (Figure 3A, lanes 6 and 7 versus lane 3). The lack of alleviation by RMI1 was not due to insufficient RMI1 because RMI1 at similar concentrations stimulates TopoIIIα-mediated decatenation by 2-fold in the absence of RPA [26]. Together, these data indicate that BLM, but not RMI1, restores TopoIIIα decatenase activity in the presence of RPA.

BLM and RMI1 synergistically stimulate TopoIIIα decatenase activity [26]. We explored the possibility that BLM and RMI1 synergistically alleviate RPA inhibition of TopoIIIα decatenation. We found that the addition of both BLM and RMI1 stimulated TopoIIIα-mediated decatenation by a remarkable ∼30-fold even in the presence of RPA (Figure 3B, lane 5 versus lanes 2 and 3). The synergistic stimulation observed here (Figure 3B, lane 5) likely reflected the maximum decatenation achievable because an increase in RMI1 concentration (Figure 3B, lane 6 versus lane 5), or the absence of RPA in reaction mixture (Figure 3B, lane 7 versus lane 6) did not further stimulate decatenation.

Alanine mutations at the conserved Leu-Leu-Thr-Asp (-LLTD) residues of RMI1 disrupt the interaction between RMI1 and TopoIIIα and ablate the stimulation of TopoIIIα decatenase activity by BLM [26]. Similarly, we found that RMI1-LLTD mutant in the presence of BLM failed to restore TopoIIIα decatenase activity above the level seen with BLM alone (Figure 3B, lane 8 versus lane 4). Therefore, even in the presence of RPA, the synergistic stimulation requires the physical interaction between RMI1 and TopoIIIα.

### RPA negatively regulates the decatenase activity of TopoIIIα-BLM-RMI1

RPA, a core member of the BLM complex, specifically stimulates BLM helicase activity [23]–[25]. Therefore, although RPA alone negatively regulates TopoIIIα decatenase activity, it is conceivable that RPA stimulates decatenation by TopoIIIα together with BLM and RMI1 when all members of the BLM core complex are present. To test this possibility, we set up reactions containing BLM, TopoIIIα, and RMI1 at concentrations that did not produce the maximum decatenation in order to capture potential stimulation by RPA. We found that instead of stimulating the activity of the complex, RPA inhibited decatenation by BLM-TopoIIIα-RMI1, in a concentration dependent manner, by more than 3-fold (Figure 3C, lanes 3 and 4 versus lane 2). These data indicate that RPA negatively regulates TopoIIIα decatenase activity even in the presence of BLM and RMI1.

### BLM alleviates EcSSB inhibition of TopoIIIα, but not of EcTop1, decatenase activity

Since EcSSB inhibits TopoIIIα decatenase activity, we asked whether BLM and RMI1 alleviate EcSSB-mediated inhibition of TopoIIIα decatenation. We found that BLM, but not RMI1, alleviated the inhibition of TopoIIIα decatenation by EcSSB (Figure 4A, lanes 4 and 5 versus lanes 6 and 7). The addition of RMI1 to a reaction containing BLM and TopoIIIα caused a synergistic stimulation of decatenation even in the presence of excess EcSSB (Figure 4A, lane 8 versus lane 2 and 3). These data indicate that BLM, in concert with RMI1, alleviates EcSSB inhibition of TopoIIIα decatenase activity, as was the case with RPA inhibition.

To assess the relative activity of BLM in alleviating RPA or EcSSB inhibition, we monitored decatenation in reactions containing either RPA or EcSSB at concentrations that reduced TopoIIIα decatenase activity to similar levels (Figure 4B, lane 3 versus lane 6). By titrating BLM into the reaction mixtures, we found that BLM restored TopoIIIα decatenase activity to similar levels regardless of whether the activity was inhibited by RPA or EcSSB (Figure 4B, lane 4 and 5 versus lane 7 and 8). Decatenation by BLM-TopoIIIα was further enhanced when RPA or EcSSB was removed from the reaction (Figure 4B, lane 9 versus lanes 5 and 8). Therefore, although BLM physically interacts with RPA [22], [24], but not EcSSB, the finding that BLM relieves the RPA- and EcSSB-mediated inhibition at similar levels indicates that the BLM alleviation does not require physical interaction between BLM and RPA.

We next tested the ability of BLM and RMI1 to restore EcTop1-mediated decatenation in the presence of EcSSB inhibition. We have shown previously that BLM mildly stimulates EcTop1 decatenase activity, although the two proteins lack direct physical interaction [26]. RMI1, on the other hand, does not stimulate EcTop1 [26]. We found that the level of EcTop1-mediated decatenation in the presence of EcSSB inhibition remained unaffected by the addition of BLM and RMI1 (Figure 4C, lanes 4–6 versus lane 3). As a positive control to show that the enzymes were active, BLM and RMI1 stimulated EcTop1-mediated decatenation when the substrates were not pre-coated with EcSSB (Figure 4C, lane 7 versus lane 2). Therefore, unlike the situation with TopoIIIα, BLM is unable to restore EcTop1 decatenase activity in the presence of EcSSB.

## Discussion

In this study, we showed that RPA non-specifically inhibits decatenation by TopoIIIα. Such inhibition is alleviated by BLM and RMI1. BLM alleviates RPA inhibition via a mechanism that is independent of BLM-RPA interaction. These findings contrast with previous demonstrations that BLM physically interacts with RPA [22] and that the interaction is important for the optimal strand-passage activity of BLM-TopoIIIα [5].

Previous studies have shown that RPA positively regulates BLM helicase activity [22], [23] and BLM-TopoIIIα dissolution activity [5]. Interestingly, we found that RPA inhibits TopoIIIα decatenase activity at a TopoIIIα∶RPA stoichiometry near 1∶1. We envisage two possible scenarios for RPA inhibition. First, since RPA physically associates with BLM-TopoIIIα-RMI1 [20], [21], the inhibition could be mediated via direct interaction between RPA and TopoIIIα causing conformational changes in TopoIIIα that reduce its catalytic activity. Alternatively, binding of RPA to ssDNA catenanes could exclude TopoIIIα from RPA-coated substrates, thereby eliminating decatenation. Although the two scenarios are not mutually exclusive, we favor the latter for three reasons. First, like RPA, TopoIIIα recognizes and interacts with single-stranded DNA [29], [30]. Exposed single-stranded regions within DNA substrates greatly enhance the DNA relaxation activity of type 1A topoisomerases, including TopoIIIα [30]–[32]. Second, even though EcSSB and EcTop1 lack physical interactions, an excess molar ratio of EcSSB disrupts EcTop1-DNA complex formation and inhibits EcTop1-mediated DNA relaxation [28] and decatenation (this study). Last, RPA readily inhibits the decatenase activity of EcTop1, the TopoIIIα homolog in *E. coli*, suggesting that the RPA inhibition is non-specific. We propose a competitive binding model in which RPA inhibits TopoIIIα decatenation by binding to the substrates and eliminating the access of TopoIIIα to ssDNA regions, a mechanism that is independent of direct protein-protein interaction between the topoisomerase and the ssDNA binding protein (Figure 5A).

The inhibition of TopoIIIα decatenase activity by RPA is intriguing given that RPA positively regulates the dissolution activity of BLM and TopoIIIα [5]. One explanation for this discrepancy is that RPA inhibition is abrogated in the presence of BLM. Indeed, BLM readily alleviated the RPA-mediated inhibition of TopoIIIα decatenase activity. Furthermore, BLM also alleviated EcSSB-mediated inhibition, suggesting that the alleviation does not require direct BLM-RPA interaction. The non-specific nature of the inhibition (i.e., that it occurs with either RPA or EcSSB) suggests a model of alleviation in which BLM-TopoIIIα simply displaces RPA or EcSSB from the catenane (Figure 5B). On the other hand, BLM did not relieve inhibition of *E. coli* TopI by EcSSB, indicating that the cognate RecQ-helicase/type IA topoisomerase pair is important for decatenation in the presence of ssDNA binding proteins. BLM shows high affinity for various DNA structures, including the fork structure present in single-stranded catenanes [33], [34]. Therefore, the DNA binding activity of BLM could drive the BLM-TopoIIIα-RMI1 complex to the substrate, making substrate recognition by TopoIIIα unnecessary in the presence of RPA or EcSSB. Then, the enhanced decatenase activity of the BLM-TopoIIIα-RMI1 complex would overcome RPA or EcSSB inhibition (Figure 5C). As is the case with a naked ssDNA catenane [26], optimal decatenation of RPA-coated catenanes is achieved when all three members of the BLM-TopoIIIα-RMI1 complex are present.

## Materials and Methods

### Proteins

TopoIIIα and RMI1 were purified as described [26], [35]. BLM was a gift from Ian Hickson (University of Copenhagen). *Ec*Top1 (M0301S) was purchased from New England BioLabs. RPA was purchased from Enzo Life Science (ALX-201-296). EcSSB was purchased from Epicentre Biotechnologies (SSB02200).

### Single-stranded DNA decatenation assay

Single-stranded DNA decatenation assay were performed as described [26]. Briefly, purified single-stranded catenane (400 fmol) was incubated with the indicated proteins in 15 µl of reaction buffer containing 50 mM Tris-HCl (pH 7.5), 40 mM NaCl, 5 mM MgCl_2_, 1 mM DTT, and 0.1 mg/ml BSA at 37°C for 30 minutes. For reactions containing BLM, 5 mM ATP was added to initiate the reactions because the stimulation of TopoIIIα decatenase activity by BLM requires ATP [26]. For the alleviation of RPA/EcSSB inhibition experiments, RPA (or EcSSB) was incubated with single-stranded catenane at 37°C for 5 minutes before the addition of the rest of the components at 37°C for another 30 minutes.
